# The pluralization of the international: Resistance and alter-standardization in regenerative stem cell medicine

**DOI:** 10.1177/0306312715619783

**Published:** 2016-02

**Authors:** Achim Rosemann, Nattaka Chaisinthop

**Affiliations:** Centre for Education Studies, University of Warwick, Coventry, UK; Department of Anthropology, University of Sussex, Brighton, UK

**Keywords:** clinical trials, evidence-based medicine, regenerative stem cell medicine, standardization, technology regulation

## Abstract

The article explores the formation of an international politics of resistance and ‘alter-standardization’ in regenerative stem cell medicine. The absence of internationally harmonized regulatory frameworks in the clinical stem cell field and the presence of lucrative business opportunities have resulted in the formation of transnational networks adopting alternative research standards and practices. These oppose, as a universal global standard, strict evidence-based medicine clinical research protocols as defined by scientists and regulatory agencies in highly developed countries. The emergence of transnational spaces of alter-standardization is closely linked to scientific advances in rapidly developing countries such as China and India, but calls for more flexible regulatory frameworks, and the legitimization of experimental for-profit applications outside of evidence-based medical care, are emerging increasingly also within more stringently regulated countries, such as the United States and countries in the European Union. We can observe, then, a trend toward the pluralization of the standards, practices, and concepts in the stem cell field.

By working to forge international standards and a harmonized regulatory environment, the pharmaceutical economy has increased international commercialization of new drugs (van Zwanenberg et al., 2011). At the core of this integration process is the global promotion of standardized evidence-based clinical practices, the methodological ‘gold-standard’ of which is the randomized controlled clinical trial (RCT) (Timmermans and Berg, 2003). Indeed, in the contemporary era, evidence-based medicine (EBM) and the distribution of internationally recognized clinical trial methodology has evolved into a massive standardization project with global dimensions (Epstein, 1996; Mykhalovskiy and Weir, 2004; Petryna, 2009). Reliance on the RCT as the central epistemological instrument in pharmaceutical research has given rise to extensive changes in local clinical research and innovation practices: the new epistemic significance of the RCT involves far-reaching adjustments of pre-existing practices, values, material environments, and administrative procedures (Keating and Cambrosio, 2011; Petty and Heimer, 2011). However, as will become clear in this article, the global move toward standardization in medical research is also a form of international stratification. The move constructs some geographically and economically situated forms of clinical research practices as more acceptable than others, which in turn creates new boundaries of inclusion and exclusion among individuals, groups, spaces, practices, values, concepts, and things (Thevenot, 2009).

EBM and the RCT, defined by scientists and regulators in high-income countries, are increasingly contested as universal standards of clinical stem cell research and practice. New hopes among patients, lucrative business opportunities, and the lack of international harmony among regulatory frameworks have given rise to transnational resistance to standardization of RCTs. In contexts of experimental, for-profit stem cell treatments, as well as of small-to-mid-sized clinical stem cell studies, physicians, scientists, and corporate organizations oppose treating the RCT as an obligatory passage-point for market approval (McMahon, 2014; Rosemann, 2013). These stakeholders seek to maximize experimental and clinical freedoms, and to minimize the controls of drug regulatory agencies and the high costs and restrictions that characterize the formal drug approval process. As we will show, developments have motivated organized, transnational networks to adopt alternative regulatory practices and research standards. These networks promote and seek to legitimize physician-based forms of clinical translation, which operate outside the RCT format and are independent of the review procedures of drug regulatory agencies. This article offers case studies of these networks, focusing on three professional societies – in China, India, and the United States – that provide for-profit, experimental stem cell therapies.

## A diversifying regulatory landscape

Transnational resistance around EBM and RCTs circulates beyond grassroots scientific activism, even to the level of state regulation. In recent years, there has been a shift from international harmonization to diversification. For instance, in 2014, Japan introduced an approval path for stem cell treatments that avoids the phase I–III clinical trial system (Lysaght, 2014). The new fast-track path enables conditional market approval on the basis of safety and efficacy tests on small numbers of patients, complemented by a five- to seven-year period of post-marketing surveillance (Cyranoski, 2013). South Korea’s Food and Drug Administration still holds onto phase I–III clinical trials, but has introduced an accelerated system that, on a global level, enables the fastest path to market approval for stem-cell–based medical products (Cyranoski, 2013; Wohn, 2012). In China and India, drug regulatory authorities have until now issued only legally limited and provisional regulations and regulatory guidelines (Viswanathan et al., 2013; Rosemann, 2013). Although Chinese and Indian regulatory agencies endorse controlled trials in stem cell research, hundreds of clinics have provided experimental treatments without evidence from systematic clinical studies (Baker, 2005; Cyranoski, 2009; McMahon, 2014). The providers of these experimental therapies have capitalized on the promissory potential of stem cells, to offer new treatment options for previously incurable diseases, and to give rise to new therapeutic markets. These uncontrolled environments of experimental applications of stem cell research stand in sharp contrast with environments in the United States and Europe, where systematic preclinical safety studies and phase I–III trials are the cornerstones of market approval procedures for medical products (Faulkner, 2012). Uncontrolled applications have been reported in more stringently regulated countries, such as Germany and the United States (McMahon, 2014), when companies have either ignored state regulation or have exploited regulatory gray areas (Cyranoski, 2013; Sipp, 2014). These kinds of uncontrolled, commerce-driven applications of stem cell research are generally dismissed in top international journals (Cyranoski, 2006; Hyun, 2010; Kiatpongsan and Sipp, 2008), as well as in official statements by the International Society for Stem Cell Research (ISSCR, 2008) (Lindvall and Hyun, 2009). Such dismissals reflect the position of the global elite of stem cell research, a group composed mainly of researchers from high-income countries.

## The emergence of transnational networks of alter-standardization

Efforts to problematize and/or abolish experimental medicine outside the RCT format have sparked vital forms of discontent, criticism, resistance, and calls for practicable alternatives. Such opposition is increasingly transnational and organized. There is not only ethical and scientific *diversification* at the level of individual institutions (e.g. mushrooming of experimental stem cell clinics), but also a gradual shift toward a *pluralization* of ‘internationally shared’ or ‘internationally recognized’ standards, practices, and concepts. Here ‘internationally shared’, ‘internationally recognized’, and ‘universal’ refer to clinical research standards, methods, and best practice guidelines that are ostensibly internationally normative. These guidelines have been defined primarily by regulators, scientists, and pharmaceutical companies from global high-income regions and underlie (in variations) the drug-licensing procedures in a large number of countries. By ‘pluralization’ we mean the creation of novel *transnational spaces of alter-standardization* – networks, institutional spaces, rules, communities of practice, and platforms of knowledge sharing and publication that endorse and validate ethical and research protocols that diverge from mainstream international scientific standards. Conceptions of *the international* are contested and *alternative* or *parallel* constructions of the international are emerging.

Transnational resistance to EBM in general and the RCT in particular is illustrated by the emergence, since 2007, of three professional societies dedicated to the development and evaluation of cell- and stem cell–based treatments: the International Association of Neurorestoratology (IANR), the International Cellular Medicine Society (ICMS), and the Stem Cell Society of India (SCSI). IANR was initiated by a clinical researcher from Beijing, in collaboration with physicians and scientists from China, Europe, India, and the Middle East. ICMS was founded by physicians and medical entrepreneurs in the United States, and currently has members from 35 countries, with international chapters in China and various countries in South and Central America.^1^ SCSI was founded by a clinical researcher and entrepreneur from Mumbai, and has close ties with IANR.

As the geographical ties of these organizations illustrate, transnational opposition to the use of RCTs as the obligatory passage-point for market approval of stem cell technologies is increasing, even in the United States and Western Europe. In a context of intense global competition over markets and technological innovation, concerns about losing out, along with increasing health-care costs and the recent economic crisis, have resulted in calls for deregulation, more flexible regulations, and new spaces of regulatory exceptions and exemptions (Cooper and Waldby, 2014; Faulkner, 2014). Moreover, stem cell controversies and regulatory changes affect regulatory debates and processes in other areas of medical research. In the United States, for instance, think tanks and lobby groups are using the case of stem cell medicine to campaign for deregulation of drug approval, research, and therapeutic practice.

## Methodology

Our research on all three organizations includes analysis of English- and Chinese-language media, including policy documents, scientific journal articles, newspaper articles, Internet websites and documents, and television. Our research on some of the organizations is based on ethnographic fieldwork by the first author, conducted between April 2010 and April 2011. The fieldwork involved: (a) interviews with 35 stem cell researchers from 21 medical institutions in mainland China and Hong Kong, including interviews with the founder of and other researchers involved with IANR and (b) participatory observation at international scientific conferences and meetings in Taiwan and Hong Kong, including presentations by the founder of SCSI.

## The pluralization of ‘international’ forms, standards, and practices

Timmermans and Epstein (2010) point out that, because standardization commonly comes with new forms of external control of individual practitioners and institutions, resistance is an integral feature of standardization (p. 60). The global landscape of clinical stem cell research and application is an example of particularly pronounced resistance to international standardization (Cyranoski, 2012b; McMahon, 2014).

The distribution of unproven, experimental, for-profit stem cell interventions is now a global industry, characterized by the emergence of complex transnational networks (McMahon, 2014; Sleeboom-Faulkner and Patra, 2011). This industry resists loss of local autonomy through standardization by challenging fundamental tenets of the EBM paradigm (e.g. sound preclinical evidence, rigorous safety studies before first-in-human trials, and market approval following successful completion of phase I–III RCTs). The industry also defends less rigorous research regimens, in order to enable rapid clinical translations and legitimize study formats beyond the RCT (e.g. cohort studies, experimental studies based on self-comparison by patients) (Huang, 2010: 130).

At stake here is a clash between an emerging, more-or-less imported form of regulatory authority and resistance from local systems of stem cell research and clinical practice. As the case of ICMS will illustrate, resistance is not only instigated in countries with low levels of or flexible forms of regulation, such as China or India, but increasingly, also in more stringently regulated countries, such as the United States. In the latter, local physicians, private clinics, and corporations strive to replicate the high level of experimental freedom and lucrative business opportunities that can be observed in less stringently regulated countries. We argue that this transnational resistance to standardization of clinical stem cell research and application has generated a rise in pluralization of ethical and scientific standards. The stepwise process of international standardization has shaped and pluralized *local* developments around experimental clinical interventions, without superseding or replacing them.

Our exploration of these forms of ‘alter-standardization’ provides fundamental insights into the implications, responses, and unintended consequences of the global distribution of EBM research standards and RCT methodology. For example, we illustrate that locally developed, alternative forms of clinical experimentation have now also begun to globalize – to circulate around the world and become embedded in new projects. While our empirical focus is stem cell research, our analysis provides new insights to the study of other emerging fields of medical research.

## Case study I: IANR

IANR was founded in 2007 by the Beijing-based neurosurgeon Professor Hongyun Huang, in collaboration with Chinese and international partners. It has attracted members from over forty countries worldwide (Huang et al., 2012). Dr Huang co-chairs the society with the orthopedic surgeon Dr Ziad M Alzoubi of Amman, Jordan, who is also the president of the Pan-Arab Spine Society. Professor Geoffrey Raisman, chair of the Neural Regeneration Unit at University College London (UCL), is the honorary president.^2^ Dr Huang is one of the first clinical researchers in China to offer experimental, for-profit stem cells and stem-cell–like interventions. Since the early 2000s, Huang has provided interventions with fetal olfactory ensheathing cells (OECs), as well as Schwann cells, to ‘several thousands of patients, from more than eighty countries’.^3^ His approach, initially celebrated as an important breakthrough by local media in China (Central Chinese Television [CCTV], 2002) was heavily criticized in 2006 (Cyranoski, 2006; Dobkins et al., 2006; *Nanfang Zhoumo* [Southern Weekend], 2006). Huang has thus continually tried to provide evidence of the safety and efficacy of his treatments, cells, and procedures. In doing so, he has become a key advocate for alternatives to the RCT (Huang, 2010). Huang has crossed the boundary between experimental, for-profit interventions and research-oriented forms of experimental applications, since his initial experiments in humans in 2001. Unlike most for-profit providers in the stem cell field, Huang and his team have published exact details of their procedures. They have also published clinical data in the form of case reports (Chen et al., 2011, 2012, 2013b; Xi et al., 2013), long-term large-N observation studies (Huang et al., 2006, 2009), small-N clinical pilot studies (Huang et al., 2008), and, more recently, a small-scale RCT (Chen et al., 2010). The 2007 inception of IANR might be viewed as an extension of Huang’s aspiration to bring attention and legitimacy to accommodating methods of bench-to-clinic translation. Huang’s aspiration is transnational, shared by diverse members of the clinical stem cell field.

The overall focus of IANR is broader than advocating for alternatives to the RCT. At the heart of the society’s activities is the development and promotion of neurorestoratology, a field that Huang et al. (2012) describe as ‘a newborn and emerging distinct discipline of the neuro-science family’ (p. S3; see also, Huang et al., 2010). Neurorestoratology, according to Huang, embraces therapeutic strategies from diverse areas of neuroscience, such as: cell and tissue transplantation; the use of biomaterials and bioengineering; neuromodulation; and, pharmaceutical and chemical therapies. These approaches aim for neural regeneration and repair by replacing damaged components of the nervous system (Huang, 2010: 15). Neurorestoratology aims toward ‘the enhancement of the translation between basic research and clinical application’ (Huang et al., 2012: S5), in order to ‘restore neurological functions in patients’ and ‘to improve their quality of life’ (Huang, 2010: 129).

IANR (2010) gathers scientists and clinicians from widespread geographical locations and disciplinary perspectives. The association provides a professional platform for exchange of knowledge, dedicated to the transfer of basic and preclinical research findings into new clinical strategies (IANR, 2010). An explicit purpose of the association is to facilitate and ‘shorten the process of bench to bedside’ translation (IANR, 2010). To promote this goal, IANR explicitly endorses the use of efficacy assessment procedures other than RCTs, including the method of self-comparison by patients.^4^ Huang et al. (2012) argue that it is a central ‘falsehood’ that ‘randomized double blind control designed studies are the only gold standard for clinical study [and that] self-comparison designed study should be ignored and neglected’ (p. S3).

IANR (2009) does not categorically reject the use of RCTs: its charter-like document, the ‘Beijing Declaration’, states that the organization ‘supports the highest standards for clinical trials to evaluate the safety and efficacy of its neurorestorative therapies’. Nonetheless, Huang (2010) claims that for many patients with neurodegenerative diseases ‘self-comparison is the best way and the simplest tool to assess the effect of a treatment’ (p. 130). Huang (2010) also claims that, with neurodegenerative disease patients, self-comparison is ‘a much better assessment method [than randomized double-blind controlled clinical trials] …, for ethical, lawful and scientific reasons’ (p. 129). Huang’s published arguments include very little detailed explanation of these strong claims. One bit of explanation is his criticism of the use of sham-operation control groups in RCTs, which he sees as ‘questionable and unethical as it inflicts unnecessary harm on controls’ (Huang et al., 2012).

IANR strongly supports physicians’ use of ‘reasonable and practical research methods to do [clinical] studies’, instead of indiscriminately following the ‘doctrinal, or rigid way’, defined in terms of EBM and the RCT (Huang et al., 2012: 130). IANR thus diverges radically from the internationally recognized clinical research protocols typically recognized by drug regulatory agencies and top international journals. This divergence has initiated a politics of resistance that not only problematizes and rejects the global dominance of EBM and RCTs in the field of stem cell medicine, but also creates, recognizes, validates, and standardizes alternative modalities of clinical research and intervention. We discuss three alternative modalities: strategic linkages with international journals, alliances with other professional societies, and professional guidelines.

### Linkages with international journals

IANR has succeeded in initiating strategic partnerships with internationally known scientists and editors of international scientific journals. The society has secured a yearly section in the US-based, internationally peer-reviewed *Journal of Cell Transplantation* (Huang, 2010: 129), and the journal’s Editor in Chief Paul Sanberg has actively promoted neurorestoratology as a novel area of neuroscience (Huang et al., 2010, 2012; IANR, 2009). The society also has formed an alliance with the *American Journal of Neuroprotection and Neuroregeneration* and describes it as the ‘second official journal of the IANR’ (IANR, 2010). In August 2013, IANR published its first issue of the open-access *Journal of Neurorestoratology*, which functions as the ‘main official journal’ (Huang, 2013: 40). IANR-related publications in these journals have included presentations of findings from basic and preclinical research, case reports from individual or small-N groups of patients, data from long-term observational studies of patients that took part in experimental, for-profit therapy, and findings from small-N controlled trials.

### The creation of alliances with other professional societies

IANR hosts international conferences, which serve as opportunities to share knowledge. By 2014, seven symposia were conducted in China, Jordan, Romania and, most recently, India. These events bring together an interesting mixture of internationally recognized and less-recognized scientists, as well as clinical researchers and practitioners from China, India, and other Asian countries, as well as the United States, Europe, the Middle East, and South America (IANR, 2010, 2011).^5^ Huang (2013) describes the intended purposes of these conferences in terms of both building community, and also sharing novel (pre)clinical strategies and results from unpublished, alternative forms of experimental clinical research. Since these alternative studies are not typically accepted by the top international journals, they would be largely invisible if not for the conferences.

As an annual conference organizer, IANR has strategically linked itself with European and Indian professional societies. Since 2011, each of the association’s yearly meetings was co-organized by the UK-based professional society Global College of Neuroprotection and Neuroregeneration (GCNN), which, like IANR, works toward development and evaluation of emerging therapeutic opportunities and concepts in neuroregeneration (Chen et al., 2013a: 547). The GCNN has two founders, Russ Pendleton and Dr Hari Shanker Sharma. Pendleton is a London-based health-care communication specialist and consultant, who, at the time of this writing, did not seem to be involved with the College. Sharma, a professor of neurobiology at the department of surgical sciences, University of Uppsala, Sweden (Chen et al., 2013a), is now a leading force in the College and also plays a key role in IANR.^6^

In 2014, during IANR’s seventh annual conference in Mumbai, India, the society formed an alliance with the newly established SCSI and its chair, Dr Alok Sharma. Sharma is a professor of neurosurgery from the Lokmanya Tilak Municipal (LTM) General Hospital of the LTM Medical College, Mumbai, and now also a member of the chair-committee of IANR.^7^ According to its Facebook profile, SCSI is a ‘national body which has been created to bring together all professionals in India involved in the field of stem cell research and therapy’, and ‘the society will play an active role in assisting and guiding various governmental regulatory bodies and other professional bodies, while also concretizing guidelines in alignment with advances that are occurring in the field’.^8^

Of the nine members listed on SCSI’s Facebook page, seven are the heads of private medical institutions or companies that offer experimental, for-profit stem cell therapies, and the remaining two are heads of private stem cell banks. This member composition suggests to us that SCSI is a lobby group of commercial providers of experimental stem cell therapies, and does not broadly represent all of India’s stem cell researchers. Our impression is supported by YouTube videos and website materials, which indicate that Dr Sharma provides experimental for-profit stem cell interventions at Neurogen Brain and Spine Institute. Neurogen is a company that markets to patients under the name Stem Cells Mumbai,^9^ offering experimental stem cell interventions for a large number of neurodegenerative diseases (Alzheimer’s disease, ataxia, autism, cerebral palsy, dementia, multiple sclerosis, muscular dystrophy, spinal cord injury, and others). A search on YouTube revealed more than 100 patient testimonies for experimental, for-profit stem cell treatments provided by Dr Sharma.

Like Dr Huang, Dr Sharma has crossed multiple boundaries between the sphere of clinical research on the one hand, and the lucrative sphere of experimental stem cell therapies on the other. Sharma openly discusses his clinical procedures, as well as the types and amounts of cells he uses. He has taken records of the treatment results of many of his patients, using standardized outcome measures and, where possible, long-term follow-up procedures. None of these results have been published in top international journals, but Sharma has used open-access, pay-to-publish medical journals to publish individual case reports (Sharma et al., 2013a, 2013b, 2013e, 2014a, 2014b), and nonrandomized open-label clinical studies (Sharma et al., 2012, 2013c, 2013d). Sharma has also published an e-book, a collection of 100 individual case reports of patients with muscular dystrophy (Sharma et al., 2011).

Like Huang’s research, Sharma’s work has been strongly criticized by researchers from the United States and other high-income countries. At a 2010 SCSI conference in Hualien, Taiwan, the first author of this article witnessed open criticism of Sharma’s methodology and data by researchers from the United States, Canada, and Hong Kong.^10^ Sharma responded by pointing out that clinical translation through EBM research standards, and the use of phase I–III trials require resources of time, money, and material biology that are often unattainable to him and other researchers. In his view, non-EBM treatments, like experimental stem cell therapies, can improve quality of life for people who are suffering.^11^ In a recent publication, Sharma (Sharma and Gokulchandran, 2009) argues for a shift from EBM to ‘practice-based medicine’, a system in which evidence for the safety and effectiveness of a treatment is gradually accumulated from experimental clinical practice:

Clinical trials are expensive. Geron spent US$ 56 million before it could embark on its historic [phase I] embryonic stem cell study this year. Outside of the pharmaceutical and biotechnology companies these sort of resources are almost unavailable. It is time, therefore, that we [reconsider] ‘evidence based medicine’ and turn to ‘practice based evidence’ so that the individual practitioner of medicine could be a part of the newer developments and evaluation of the systems of medicine. Ninety percent of current neurosurgical practice is not supported by prospective randomized double blind clinical trials. … Progress in medicine has come when individual physicians pioneered newer form of therapy that they believed in. Day to day decisions made in clinical practice, specially in intensive care setups and operating rooms are made empirically, [and are] based on the treating physicians experiences and approach, and the clinical circumstances at hand. … Evidence generated from the individual physicians practice needs to be respected too. Thus ‘practice based evidence’ needs to be looked at in a way similar to ‘evidence based medicine’. (pp. 9–10)

Sharma’s advocacy for alternatives to EBM is based on reasoning analogous to the reasoning behind IANR’s advocacy for alternatives to the RCT.

### The development of professional guidelines

IANR and SCSI have both appealed to paragraph 35 of the Declaration of Helsinki (e.g. Huang, 2010). Although the declaration states that unproven or new therapeutic measures can be provided to critically ill patients when existing therapeutic methods have been ineffective or do not exist, it is an open question whether paragraph 35 originally referred to for-profit, experimental treatments for thousands of patients. However, Huang and IANR have tried to legitimize their work by other means than claiming to help seriously ill patients. For example, the Chinese Branch of IANR has published the ‘Standard Recommendations for the Application of Chinese Clinical Cell Therapy’ (Huang, 2013), a statement of standards and guiding principles for evaluating the safety and efficacy of an experimental intervention. The document acknowledges the lack of a consistent regulatory framework for stem cell research and therapeutic applications in China:

[The] application [of cellular therapy] for neurological diseases currently lacks worldwide consensus standards. China is one of the pioneer countries conducting this therapeutic technology and is leading the development of this frontier. Chinese physicians have applied cell therapy to a variety of intractable neurological diseases and contributed to the establishment and development of the theoretical system of neurorestoratology. Therefore, it is our responsibility to standardize the clinical procedures for promoting the application of cell therapy as one strategy for treating neurological disorders. This will greatly contribute to the international community supporting cell-based neurorestorative therapy. (Chinese Branch of IANR, 2013)

The document is clearly an attempt to enhance the credibility of experimental cellular therapy providers and the claims to safety and efficacy of available experimental treatments. In the light of domestic criticism of experimental, for-profit stem cell interventions in China by the media, scientists, bioethicists, and policy makers (Rosemann, 2013), the development of professional standards and guidelines by IANR can also be read as an attempt to distinguish members of IANR from other providers of experimental stem cell interventions whom they deem highly irresponsible or fraudulent.

## Case study II: The ICMS

In 2009, two Americans, David Audley and Dr Christopher J Centeno, established the ICMS. According to its website, the society ‘represents physicians and researchers from over 35 countries who share a mission to provide scientifically credible and medically appropriate treatments to informed patients’.^12^ Audley is a business development specialist who served as ICMS executive director until 2012.^13^ Centeno is a specialist in physical medicine, interventional orthopedics, and rehabilitation.^14^

Centeno is also the co-owner of the Centeno–Schultz Clinic, a clinic in Colorado that offers experimental therapies with autologous mesenchymal stem cells to treat various orthopedic conditions.^15^ These therapies are based on nonsurgical injection procedures that were developed by Regenexx Inc., another company whose majority shareholders are Centeno and his partner John R Schultz (Barrett, 2012). According to the Regenexx website, the company’s autologous stem cell procedure is available at twenty clinics all over the United States.^16^ Like Huang’s treatments, Regenexx’s treatment has been praised by some^17^ and fiercely criticized by others.^18^ In 2008, Regenexx received a warning letter from the US Food and Drug Administration (FDA), stating that the company’s experimental use of cells as a drug without an FDA-issued biologics license was in violation of the law (Cyranoski, 2010; Malarkey, 2008). The FDA filed a lawsuit against the company in 2010. In 2012, a US district judge granted a permanent injunction against application of the company’s stem cell procedure in the absence of demonstrated compliance with FDA regulations (Barrett, 2012).

While Regenexx has appealed the ruling (Barrett, 2012) and has continued to offer autologous mesenchymal stem cell treatments in affiliated clinics, according to a media briefing posted on the company’s website, all ‘procedures currently offered [by Regenexx] in the US are […] not considered a 351 drug [i.e. a biologics drug product] by [the] FDA’ and thus fall out of the legal reach of the FDA.^19^ As per a Regenexx media release, ‘the only Regenexx procedure considered a drug by FDA was the Regenexx-C procedure, which has not been performed in the US since 2010’,^20^ but is still available via a facility in the Cayman Islands.^21^ Via a subsidiary company, called Neostem, Regenexx seems to license its stem cell procedures in China, as well.^22^

In the string of events preceding the FDA’s lawsuit, Centeno stated repeatedly that he welcomed the trial because it offered an opportunity ‘to formally question the FDA on its policy’ (Koleva, 2012). In a Forbes article, he was cited as claiming that the case formed a ‘21st century civil rights issue that will define what control [patients] have about the use of [their] own cells and tissue’ (Koleva, 2012). Even after losing the case, Centeno and Regenexx’s struggle to ‘move stem cell therapy into the mainstream’ (cited in Sternberg, 2011) continues.

As indicated in a *USA Today* article, the inception of ICMS is an integral part of Centeno and Regenexx’s struggle (Sternberg, 2011). According to Audley, now the former Executive Director of ICMS, the Society has grown from a ‘handful of physicians’ in 2009 ‘to an organization with over 3500 members from over 35 countries’ by 2013.^23^ ICMS is registered as a nonprofit professional organization in the United States, with international chapters in China, Argentina, Peru, Mexico, and Venezuela.^24^ A central objective of ICMS is to ‘support legitimate medical innovation outside of the context of a formal clinical trial’.^25^ As the Society’s co-founder and former President, Centeno speaks of the ‘need for dual therapy discovery pathways’, a system outside of the RCT process and outside of the review procedures of the FDA, in which a ‘physician innovation pathway’ parallels approval by the FDA (Centeno, cited in Knoepfler, 2012b). The main focus of ICMS is to promote and legitimize physician-led experimental treatments with autologous adult stem cells, which, under current FDA regulation, are regulated as a drug product, at least if the cells are more than minimally manipulated (Malarkey, 2008). The current President of ICMS, plastic surgeon and owner of a Baltimore private clinic, Dr Ricardo Rodriguez, writes:

Autologous therapy is subject to the same FDA regulations imposed for [the] mass manufacturing of drugs. … At the moment, anyone trying to jump ahead of the FDA will face serious consequences. … The problem as I see it is that adult autologous cell therapy is a highly individualized therapy. As such, the cost of going through an IND [investigational new drug] and BLA [biologics license application] process will make it prohibitively expensive to bring to market, as the cost of developing the therapy is not amortized by mass production. (Rodriguez, in Knoepfler, 2012a)

According to Centeno, physician-based forms of clinical translation are more viable ways of moving autologous stem cell treatments to the clinic, because they are not only less costly, but also faster. However, he admits that ‘the physician pathway for innovation … is hampered by lower quality data’ regarding the efficacy and safety of an experimental treatment (Centeno, in Knoepfler, 2012b).

### The development of alternative standards and self-regulatory practices

To facilitate both the production of more reliable data, and also safer and more responsible use of experimental stem cell applications, ICMS has developed its own professional guidelines, an institutional review board (IRB) service, an open registry of stem cell treatment outcomes, and a stem cell accreditation program.

ICMS professional guidelines are ‘intended to provide a framework for the clinical translation of cell-based therapies’,^26^ and refer to ‘guidelines for the practice of cell-based medicine’, in relation to physician-led forms of medical experimentation.^27^ These guidelines include a range of minimum standards for the experimental application of stem cell therapies, and detailed instructions on issues related to patient recruitment, clinical practice, laboratory practice, the re-implantation of cells, record keeping, and patient care.

ICMS’ IRB is composed of ICMS-affiliated medical practitioners and researchers.^28^ Physicians and clinics (including commercial providers of stem cell therapies) may present experimental treatment protocols to the IRB, to be evaluated in terms of patient safety. Approval of proposed procedures is based on ICMS’ guidelines.^29^ The ICMS IRB continues to monitor the progress of the reviewed application once approved, and clinics are admonished to report and document all serious adverse events for further evaluation.^30^

The ICMS Open Treatment Registry is a database that stores treatment outcomes and complications of stem cell treatments, as reported by patients who have undergone experimental stem cell therapies (ICMS, 2010a: 5).^31^ ICMS reportedly conducts a follow-up questionnaire, with each ‘patient at 3, 6 and 12 months and then at 2, 3, 4, 5, 10 and 20 years’. Follow-up information is collected online (via emails and a website), by telephone and by mail (ICMS, 2010a: 6). Storage of patient records involves a contractual agreement between ICMS and individual stem cell clinics. Participating patients pay a fee of US$350 (ICMS, 2010a: 6). In March 2011, ICMS reported that its Open Treatment Registry stored over 750 adult stem cell patient cases.^32^ On its website, ICMS claims that the number of patients has since grown to 1100 patients. However, the registry is only accessible to members of ICMS, so we did not have access to the current number of patient cases, nor to data or treatment results. To include data from additional patients in the registry, ICMS collaborates with stem cell clinics and companies that meet the ‘society’s minimum standards and [that have] been reviewed by the ICMS Institutional Review Board’.^33^ One of these companies is the Korean corporation RNL Bio, whose patients have contributed to the ICMS’ registry since 2010.^34^ As stated in an online information brochure, clinics that participate in the association’s Open Treatment Registry are able publicly to advertise their participation, and ICMS (2010b) acknowledges that ‘advertising that an independent nonprofit is providing patient outcome tracking is a powerful marketing tool for clinics’ (p. 5).

ICMS’ stem cell clinic accreditation program provides evaluation services to clinics that offer experimental stem cell applications, focusing on clinic treatment procedures, informed consent protocols and patient documentation and recruitment strategies.^35^ The program also addresses the ‘clinic’s cell collection, processing, and implantation practices’, according to ICMS’ ‘Best Practice Guidelines’. The records of all patients treated by ICMS-accredited clinics are stored in the ICMS’ Open Treatment Registry, and are used to assess treatment outcomes and complications.^36^ The ICMS’ accreditation process takes eighteen months, without involvement of drug regulatory authorities, and independent of the regulatory requirements of the countries in which a stem cell clinic is located. Accreditation of a clinic does not require data from controlled clinical trials. On 30 May 2012, it was announced that the American Association of Blood Banks (AABB) had entered into a partnership with ICMS, with the purpose of ‘assisting the ICMS in the development of quality-based, voluntary standards for the collection, processing and administration of autologous adult cells’, as well as to ‘provide global accreditation services against the ICMS standards’ to stem cell clinics, so as to ‘foster responsible innovation and promote patient safety’.^37^ Reports from May 2011 indicate that two private medical organizations participated in the ICMS’ Stem Cell Clinic Accreditation Program:^38^ the Regenerative Medicine Institute (RMI) and World Stem Cells LLC. RMI offers for-profit stem cell therapies in Tijuana, Mexico, at the private Angeles Hospital, and recently has begun to do patient-funded clinical stem cell studies of chronic obstructive pulmonary disease and heart disease;^39^ in February 2012, ICMS announced via its website that its first accredited medical facility was the RMI.^40^ World Stem Cells LLC operates with a contract clinic in Cancun, Mexico, offering experimental stem-cell–based procedures to patients with more than 31 disease conditions, ranging from Alzheimer’s disease to ulcerative colitis.^41^ Like other providers of unverified for-profit stem cell treatments, the company advertises its treatments with individual patient testimonies.^42^

## Differences between ISSCR and ICMS standards

At a surface level, ICMS’ standards and self-regulatory practices involve interesting parallels with the regulatory guidelines and instruments of medical societies that are committed to EBM standards, such as ISSCR and its *Guidelines for the Clinical Translation of Stem Cells* (ISSCR, 2008). Like the ISSCR document, the ICMS guidelines emphasize the need for both ongoing IRB reviews of investigational treatments, as well as evaluations by an independent oversight body. Furthermore, both organizations insist on systems for monitoring the long-term health effects of administered treatments, for reporting adverse events, for making medical claims based on acknowledged scientific evidence, and for ensuring quality and safety of procurement, storage, processing and transplantation of cells, to minimize the risk of contamination, infection, and other health risks (ICMS, 2010a).

Despite these similarities between ICMS and ISSCR, the organizations differ significantly in terms of clinical methodologies, types of evidence, and lines of ethical reasoning (cf. Blasimme, 2013). While the ICMS guidelines endorse medical innovation outside of formal clinical trials, the ISSCR document calls for evidence-based clinical trials and market approval procedures that follow a ‘cells-as-drugs’ approach based on rigorous regulatory oversight (Blasimme, 2013). ISSCR regulation rests on the adaptation of existing regulatory categories for the governance of cell-based technologies and the use of the phase I–III RCT system. It calls for ‘many years of preclinical research and clinical testing prior to the maturation of experimental treatments into an accepted standard of medical practice’ (ISSCR, 2008).

The forms of clinical evidence that ICMS (2010a) promotes are based on a clinical staging process that mimics the epistemic structure of stepwise clinical trials. According to this scheme, a cell type that has never been used in humans but for which promising data from preclinical studies exist is first tested in a small number of patients (5–10) before being used in a larger cohort of patients (20–50). If no significant complications are reported during a 6-month follow-up period, and if patients show some signs of improvement, the cell type can be applied to a larger number of patients (up to 300). Treatment of these patients is followed once more by an evaluation period of 6 months, during which physicians should ‘be able to document subjective and objective outcome measures’ (ICMS, 2010a: 7). After this, a tested cell type ‘has completed all stages’ and can be ‘used in patients in an unrestricted fashion’ (ICMS, 2010a: 8). However, treated patients must be entered into the above-mentioned ICMS registry for follow-ups and long-term tracking of treatment efficacy and safety (ICMS, 2010a: 20; cf. Blasimme, 2013). ICMS’ system thus regulates experimental medical procedures proportionate to risks in a way that parallels the approaches of regulatory agencies and ISSCR (Blasimme, 2013: 36). However, ICMS deviates from the phase I–III RCT system: the types of clinical studies and evidence ICMS promotes do not involve the systematic use of control groups, randomization, or the blinding of administered treatment procedures. Because most of the clinics that request IRB or accreditation services from ICMS are private hospitals or companies that offer experimental stem cell treatments to patients on a for-profit basis, participation in these open-label studies can be expected to be based on a pay-to-participate schema. Moreover, unlike ISSCR and drug regulatory agencies, ICMS has no legal power to enforce its standards and guidelines in the hospitals that make use of its IRB and accreditation services.

With its efforts to establish shared conventions and standardized modes of knowledge production through a collective program of action, ICMS reproduces important aspects of the epistemic machinery of the EBM system (Cambrosio et al., 2009). However, rather than aiming to replace the forms of objectivity that RCTs and EBM standards generate, the alternative standards of ICMS aim to produce ‘public guarantees’ (Thevenot, 2009) that enable and legitimize a ‘parallel space’ of therapy production outside of the legal and epistemic authority of regulatory authorities such as the FDA.

## Responses to ICMS

The activities of ICMS have been met with mixed reactions. Scientists and commentators with ties to ISSCR typically have rejected outright ICMS’ attempts to sideline the review procedures of drug regulatory authorities. Doug Sipp, for instance, a former member of ISSCR’s task force on unproven stem cell therapies, has argued that stem cells should be introduced ‘into the clinic only via responsible, rigorous and ethical scientific testing’ (Sipp, in Knoepfler, 2012b). According to Elaine Fuchs, the former president of ISSCR, rigorous testing is a high benchmark, but necessary to protect vulnerable patients (Dolgin, 2010). Opponents of ICMS have questioned the scientific integrity of the suggested review and accreditation procedures, and have emphasized the organization’s close ties with commercial stem cell clinics. An article in *Nature*, for instance, has labeled ICMS a ‘stem cell lobby-group’ and has expressed concerns that members of ICMS put their own financial interests ahead of their patients’ health interests (Cyranoski, 2012a). The US FDA has also raised concern that there are conflicts of interest in ICMS’ IRB services. The FDA (2012) audited the IRB in 2012, reporting that the services should be run more strictly and be offered exclusively for clinical applications that ‘are exempt from FDA regulation’. The FDA (2012) also reported that the IRB had approved a study in 2011 that was subject to FDA regulation (because it used cells that were more than minimally manipulated), and that had been put on a hold by the FDA in 2009.

Others have taken a more accommodating view of ICMS. Bernard Siegel, spokesperson for the Stem Cell Action Coalition and director of the Genetics Policy Institute in Florida, has argued that ICMS is ‘not just … created to hype this entire field’, but is an organization that is actually trying to look at for-profit stem cell clinics and to offer a perspective to regulate these hospitals (Siegel, quoted in: Dolgin, 2010). Paul Knoepfler (2012c), a stem cell lab leader and science writer at the University of California Davis, comments that even though ICMS is ‘viewed somewhat skeptically by the stem cell community, … they are undeniably an important player in the stem cell world’. While Knoepfler (2012e) supports ‘the FDA mission for safe and effective therapies’, he acknowledges that ‘there’s literally millions of patients who don’t have any option right now based on today’s medicine, and naturally looking for other options’. Like Siegel, Knoepfler recognizes that ICMS offers forms of observation and peer oversight of clinics in many countries where clinics were previously completely uncontrolled. Knoepfler (2012d) sees, however, the need for improvements to ICMS, such as an ‘external advisory board of leaders … who are not members of ICMS’, and ‘a greater level of openness’, so that ‘patients and researchers can sit down together and have an open and critical discussion, where patient’s voices can be heard’.

Still others have publicly lauded the society’s efforts to increase the space for physician-based innovation in the stem cell field. Barbara Hanson (2010), for instance, the co-founder of the patient-moderated online forum *Stem Cell Pioneers*, posted: ‘we need something right now, and we need practical advice, and this is what the ICMS is providing’. Hanson refers to widespread demands for more information about stem cell clinics, from patients who want to make better-informed decisions and to join experimental stem cell treatments. Similar opinions have also been expressed from other patient groups. The organization Patients for Stem Cells, for instance, has stated that it ‘appreciates the efforts brought forth by the ICMS, to have more open and honest debate’ regarding cell and stem cell therapies (Ziegler, 2013).

## The repercussions of alter-standardization

With the development of clinical guidelines, new forms of ethical review, and novel platforms of data and knowledge sharing, organizations such as ICMS, IANR, and SCSI have started to intervene into a gray area of experimental practices that, due to its sheer size, is difficult for drug regulatory authorities to control. While the self-regulatory activities of ICMS, IANR, and SCSI are characterized by conflicts of interest, these organizations have started to change existing experimental practices in ways that make them more accountable to patients. However, because adherence to these organizations’ regulatory standards is not mandatory, it is difficult to say to what extent and in what ways these standards transform actual clinical research and applications practices. In this article, we can only provide some details about ICMS. Additional fieldwork would be required to make further claims about the repercussions of IANR’s and SCSI’s regulations.

In 2012, at the time the FDA audited the IRB services of ICMS, the organization had reviewed protocols for at least 29 clinics and companies. Since then, ICMS has continued to offer its IRB services, among others to the Cell Surgical Network, reportedly the largest US-based, for-profit network of clinics offering autologous stem cell treatments (Knoepfler, 2013). According to Paul Knoepfler, who is a key observer of the for-profit stem cell sector, ICMS administers not one, but several IRBs that serve individual clinical organizations and networks. The Cell Surgical Network, for instance, had its own IRB that was managed by ICMS and that operated on the basis of ICMS guidelines.^43^ ICMS’ clinical accreditation program has advanced at a smaller rate. By May 2012, fifteen clinics were reportedly at various stages of the accreditation process (Rodriguez, 2012). In the same year, ICMS entered a partnership with the AABB and announced a global joint-accreditation program.

Aside from its regulatory efforts, a key activity of ICMS has been to mobilize support among peers, academics, and government officials (Rodriguez, 2012). In its struggle to use and regulate autologous adult stem cell treatments independent of FDA controls, the organization has received backing from various sides. Richard A Epstein (2013), for example, a legal scholar from the Chicago Law School and a libertarian critic of the current FDA premarketing testing model (cf. Cooper and Waldby, 2014), has publicly endorsed ICMS’ attempts to maximize physician-based innovation outside of the review system of drug regulatory authorities. According to Epstein (2013), the FDA’s regulation of stem cell procedures is misguided, and an example of administrative overreach that blocks medical innovation, and prevents access to potentially helpful treatments. Support for ICMS has also come from elected officials. Berkley Bedell (2012), for instance, a former congressman from Iowa and well-known advocate for health freedom, has argued that ICMS plays an important role in promoting physician innovation and in advancing autologous stem cell treatments in a more effective and quicker way than the FDA.

It is important to understand that these examples of support reflect a broad activist movement in the United States that is concerned with far more than stem cell research and the activities of ICMS. Epstein and Bedell, for instance, are both long-standing critics of FDA policies and campaign for decreased regulation and wider availability of investigative drugs (Cooper and Waldby, 2014). The debate on stem cell treatments has been taken up in a long-standing battle for deregulation, the maximization of experimental freedoms and increased choices for patients. Several US think tanks, for example, have used stem cells to promote ‘free to choose medicine’ or ‘right-to-try’ legislation, which offer patients and physicians the choice to use not-yet approved drugs after preliminary forms of safety and efficacy testing (Bianco and Sipp, 2014; Darrow et al., 2015; Madden and Conko, 2013). Four US states have recently passed right-to-try bills that allow for the use of medicines that have undergone basic safety tests (Rudavsky, 2015). These activities have resulted in a Federal bill – the Compassionate Freedom of Choice Act – that was put forward to the US congress in April 2014. According to this bill, investigational stem cell technologies could be manufactured, imported, distributed, and sold to terminally ill patients at a national level, without any form of intervention or restriction by the FDA (Morgan, 2014). Stem cell scientists and organizations such as ICMS, IANR, and SCSI may have been wittingly or unwittingly drawn into this struggle, but the fact is that the clinical use of cells and stem cells has started to play a key role in a wider conflict for decreased regulation and the re-articulation of the forms, boundaries, and limitations of the EBM paradigm.

## Discussion

The ‘quality’ of clinical research data and associated potential for translation is usually assessed along an epistemic hierarchy, with RCTs on top and less systematic clinical research methods, such as self-comparison studies, or expert consensus based on individual case reports, at the lower end (Thevenot, 2009). In the global landscape of regenerative stem cell medicine, however, this hierarchy – and the role of RCTs as an obligatory passage-point for routine clinical applications – has been contested. A transnational politics of resistance has emerged. The formation and activities of societies such as IANR, ICMS, and SCSI constitute a strategic attempt to revalue, legitimize, and garner recognition for physician-based forms of clinical experimentation. These organizations have produced alternative systems of research standardization and validation, which are stabilized by the creation of new types of cross-national alliances, the development of guidelines and accreditation systems, and the instigation of novel platforms for global research communication and publication. As we have shown, these alternative standards are promoted through the publication of guidelines, opinion pieces in medical and scientific journals, international conferences, blog contributions, and, in the case of ICMS, through the provision of its review and accreditation services.

From a historical perspective, there is nothing new about resistance to EBM standards or attempts to legitimize the commercial use of medical technologies prior to the existence of systematic proof of efficacy, safety, and value in patient care. As Blume (2010) has shown, many areas of medicine provide new treatments ahead of reliable evidence. However, the forms of resistance and alter-standardization that have emerged in the stem cell field go far beyond attempts to legitimize controversial medical practices that are offered by profit-oriented researchers, physicians, or medical entrepreneurs. What is at stake is a fundamental change in the regulation of cell-based technologies on a global scale, with potential implications for the regulation of other tissue-engineered products and new molecular entities. This process is exemplified not just by the alter-standards and self-regulatory practices that have been described in this article, but also by the increasing level of regulatory divergence in the stem cell field across countries and the important role that stem cells play in the struggle for ‘right-to-choose’ legislation and deregulation of drug approval procedures. The recent regulatory sea change for cellular therapies in Japan in particular (which has completely abandoned the phase I–III RCT system) indicates that the influential position of large-scale RCTs – at least in the stem cell field – is partially re-defined.

### Factors that enable alter-standardization in the stem cell field

What are the underlying reasons for this partial success of alter-standardization? In particular, how do the structural features of stem cell research enable actors to construct alternative regulatory practices and standards, setting the field apart from other areas of medical research? We suggest four factors that enable alter-standardization in the stem cell field: unique categorical challenges of stem cell medicine to regulatory agencies, high economic and therapeutic hopes, current regulatory void, and the nascent developmental stage of stem cell medicine.

First, stem cells pose a unique challenge to regulatory agencies: ‘[S]tandard pharmaceutical paradigms do not wholly apply and accordingly, [and] stem cell therapies do not neatly fit into current regulatory categories’ (Martell et al., 2010: 451). As a result of the specific biological and technical characteristics and risks of stem cell treatments, regulatory frameworks have emerged only gradually, and often in highly divergent ways across countries. This divergence has given rise to uncertainties and has prevented effective forms of international harmonization, especially outside of North America and the European Union (Hogle, 2014). Moreover, as we have shown above, this high level of regulatory variation has enabled very different modalities of clinical translation and widespread availability of experimental for-profit treatments. Experimental stem cell treatments have evolved into a lucrative industry, not only in countries with minimal or lenient regulations, but also in the United States and other stringently regulated countries (where clinics and companies have used regulatory loopholes for autologous stem cells) (McMahon, 2014). The growing numbers of clinics and transnational corporate infrastructures that offer these treatments have made it increasingly difficult in some countries for governmental agencies to intervene. The effective regulation of these experimental treatments is expensive, staff-intensive, and often beyond the administrative capacities of regulatory authorities. Furthermore, experimental stem cell therapies are widely tolerated in India, China, and other countries where regulatory frameworks are permissive and only gradually emerging. In China, for example, high-ranked politicians and the media have repeatedly praised the activities of large private enterprises in the stem cell sector (that have generated massive profits from experimental for-profit therapies) (Rosemann, 2013). These companies have been described as important drivers for national medical innovation and economic progress (Sleeboom-Faulkner, 2014).

Second, acceptance of non-EBM experimental stem cell applications is intimately linked to high economic and therapeutic hopes (McMahon, 2014). In government policies and the media, stem cells have for many years been described as a key technology to treat some of the world’s most vexing diseases, to reduce health-care costs, and to create new forms of economic revenue (Cooper, 2008). In many countries, permissive regulations and the development of less stringent forms of clinical validation and standards are associated with national economic and health-care advantages, as well as competitive advantages (Sleeboom-Faulkner, 2014). For example, in Japan there has recently been regulatory reform around stem cell therapies (especially the Japanese-invented iPS cells), which aims to aid in the translation of stem cells into routine applications more rapidly and more cost-effectively than in other countries. Japan’s abandonment of the phase I–III RCT system for stem cells will have far-reaching effects on other countries. In China, where regulations for the clinical use of stem cells are still in preparation, many researchers and bioethicists have stated that strict regulations – as handled in the United States and Europe – would stifle scientific progress, reduce economic opportunities, and prevent potential benefits for patients (Rosemann, 2013). The more permissive regulatory circumstances in these and many other countries are – from this perspective – an integral part of national development strategies (Sleeboom-Faulkner et al., submitted). These strategies accept less rigorous forms of evidence – taking on higher health and reputational risks – in exchange for a greater level of experimental freedom and the promise of more rapid economic revenues. In Japan and other countries where alternative regulatory practices for cellular therapies become integrated into formal market approval procedures, these strategies turn into a constitutive element of the experimental machinery of biocapital (cf. Rajan, 2010).

Third, the self-regulatory efforts and alternative guidelines, review procedures, and standards of organizations like IANR, ICMS, and SCSI promise to address an important regulatory void and seem to offer at least some forms of oversight and control that might mitigate risks and prevent fraudulent treatments. Even in the most flexibly regulated countries such as India, China, or Mexico, it has become clear to regulators that the mass lack of regulation of experimental stem cell treatments is dangerous, because it may result in ‘snake-oil’ applications, health hazards, dissatisfied citizens, and reputational damage. It is for this reason that – especially in developing countries – interest in the self-regulatory activities of ICMS and IANR has been most pronounced. David Audley, the former president of ICMS, for instance, claims that he has ‘actively worked with governmental officials in South America, Asia and the Caribbean to develop local standards for the evaluation of cell-based medical therapies’.^44^

Fourth, in contrast with more established forms of medical research with chemical compounds and small molecules, regenerative stem cell medicine is still at a relatively early developmental stage. Investments from the pharmaceutical industry have remained small, and cross-border marketing of stem cell–based products and procedures is still at a low level. For this reason, external pressure for international harmonization of clinical stem cell regulation has not been very strong. This situation stands in contrast with the situation of pharmaceutical drug research. The pharmaceutical industry, along with the drug regulatory authorities of high-income countries with large drug markets, has enforced international regulatory harmonization.

### The geographic connotations of alter-standardization

Resistance and alter-standardization in stem cell medicine have important geographic dimensions. On the one hand, the development of alternative standards and practices is closely related to scientific advances in rapidly developing countries such as China and India. The availability of new types of financial, technological, and infrastructural resources in these countries has enabled novel possibilities of clinical innovation, and has led to the development of new types of medical treatments and services, including experimental for-profit interventions that are prohibited in other countries. On the other hand, growing support for alternative forms of clinical translation in stem cell medicine is linked to significant regulatory differences between countries, especially the tendency in developing and economic threshold countries to regulate promissory forms of experimental research in minimal and permissive ways (see McMahon, 2014; Sleeboom-Faulkner and Patra, 2011).

In different geographic regions, very different kinds of public and private institutions and funding support stem cell innovations, differently shaping and prioritizing modes and speeds of clinical translation and commercial applications (cf. Thompson, 2010). Emergent clinical applications, and their epistemic and organizational pathways, are no longer bound to isolated localities, but have started to globalize through new transnational networks. These developments involve significant reconfigurations of global innovation landscapes, and infrastructures that are manifest in important changes in the global flows that constitute, commercialize, regulate, and apply bioscientific inventions (Thompson, 2010).

This combination of new scientific capacities and lenient regulatory frameworks in many of the emerging global center regions is likely to have important global repercussions. More rapid forms of profit-making, and the existence of less restrictive and less costly regimes of control, are likely to result in calls for more relaxed regulation within highly developed countries. In the United States, for example, widely visible political actors have promoted more lenient regulation for the provision of experimental for-profit interventions with stem cells. Governor Rick Perry in Texas, for instance, has actively lobbied for less stringent regulatory controls of stem cell therapies. The Texas Medical Board proposed a draft policy to formally approve experimental for-profit applications with autologous stem cells in local hospitals, outside of the controls of the FDA (Cyranoski, 2012b), although a subsequent court ruling undermined this proposal (Ackerman, 2012).

## Conclusion

The societies we have discussed have garnered support for physician-based forms of clinical for-profit experimentation by forming novel transnational spaces of alter-standardization. They have forged strategic alliances with scientific journals, internationally renowned scientists, and other professional societies, and they have created new databases and platforms for sharing knowledge. These activities occur at the fault-lines of the global diffusion of EBM and RCTs, responding directly to exclusion of alternative stem cell medicine. Vital forms of resistance and the creation of alternative systems of research standardization accompany efforts to mobilize EBM as the global standard in stem cell medicine.

Forms of resistance have become increasingly transnational and organized; there has been gradual pluralization of the categories, standards, and practices designated as ‘internationally shared’ and ‘internationally recognized’. Professional societies such as IANR, SCSI, and ICMS play a pivotal role in this transnational politics of resistance. Using tools like conferences, publications, international networks, professional guidelines, and accreditation procedures, these organizations have gradually transformed previously marginalized forms of experimental clinical research, making what was once unacceptable an increasingly tolerated component of the ordinary. This process is characterized by renegotiation of the limits between the recognized and the unacceptable in the international arena of clinical stem cell medicine.

As alternative professional societies adopt guidelines and review and accreditation procedures, practices of non-EBM are reordered and experimental cell and stem cell treatments are standardized, making them more transparent and accountable. However, because alternative professional societies have no legally binding power to enforce their standards and guidelines, it is likely that the integrity of alter-standardization varies in different institutions. A more nuanced view of the effects of these societies would require more research on the implementation of their standards.

Emerging forms of resistance and the development of alter-standardization are shaped by competition and global asymmetries – differences in wealth, medical education, health care, and differentiated access to financial, technological, and infrastructural resources (cf. Sleeboom-Faulkner, 2013). As this article has illustrated, efforts to legitimize forms of clinical translation outside of the RCT format are closely linked to highly stratified structures of access to the financial and infrastructural resources requisite for rigorous phase I–III trials. Conflicts around experimental practices, technology regulation, standards, and professional guidelines are understudied aspects of wider and more complex dynamics of global competition around new economic opportunities, employment possibilities, and scientific breakthroughs. To further explore the significant problem of global competition, it will be of particular importance to focus on advances in rapidly developing countries such as China, India, and other countries in Asia. As this article has indicated, these emerging scientific center regions are the loci of resistance to standards defined by elite scientists and policy makers in high-income countries.

The concept of alter-standardization enables us to situate the stem cell field in relation to other forms of science, medicine, and technology. If we continue to trace the controversies of EBM in stem cell medicine and to map concomitant transnational spaces and communities of alter-standardization, we can clarify the implications of these developments for processes of international harmonization, the implementation of national regulatory frameworks, and the planning and conduct of international clinical research collaborations.

## Notes

1. Available at: http://www.cellmedicinesociety.org/ (accessed 4 November 2015).

2. Available at: http://ianr.org.cn/english/zhuang.asp (accessed 4 November 2015).

3. Available at: http://www.nrrfr.com/E/iframe.asp (accessed 4 November 2015).

4. Self-comparison is the cross-case documentation of treatment efficacy, on the basis of before versus after treatment comparison by patients themselves; it is mainly based on subjective experience and anecdotal evidence.

5. For an overview of the programs of all annual International Association of Neurorestorato-logy (IANR) conferences, see: http://ianr.org.cn/english/xueshu.asp?id=145,146 (accessed 4 November 2015).

6. The IANR website describes Dr Shanker Sharma as the society’s elected president, and the director of research of IANR, for central nervous system (CNS) disorders and nerve repair. URL: http://ianr.org.cn/english/zhuang.asp (accessed 4 November 2015).

7. Profile of Dr Alok Sharma on the IANR website: Available at: http://www.ianr.org.cn/english/fc.asp?id=70,143 (accessed 4 November 2015).

8. Available at: https://www.facebook.com/2014.SCSI (accessed 4 November 2015).

9. Affiliation of Dr Sharma with Neurogen: Available at: http://www.neurogen.in/index.php?option=com_content&view=article&id=6&Itemid=8; website of Stem Cell Mumbai: Available at: http://www.stemcellsmumbai.com/ (accessed 4 November 2015).

10. This conference was the ‘SCI Workshop – Barriers to Cure’, organized by the Bedford Center for Stem Cell Research, Massachusetts, USA, and the China Spinal Cord Injury Network, held from 21 to 24 April 2010, at the Tzu-Chi Medical Campus, Hualien. Taiwan.

11. These statements are based on stenographic notes made by the first author during Dr Sharma’s speech, at the final panel of the Hualien Conference, on 24 April 2010.

12. Available at: http://www.cellmedicinesociety.org/ (accessed 4 November 2015).

13. Available at: http://www.linkedin.com/in/dbaudley (accessed 4 November 2015).

14. Available at: http://www.linkedin.com/pub/chris-centeno/24/380/b68 (accessed 4 November 2015).

15. Available at: http://www.centenoschultz.com/services/regenexx-procedures/ (accessed 4 November 2015).

16. Available at: http://www.regenexx.com/find-a-physician/ (accessed 4 November 2015).

17. Positive reactions to Regenexx’s stem cell therapy: (1) 5280 Magazine Available at: http://www.5280.com/magazine/2011/08/power-play (accessed 4 November 2015). (2) ABC News Available at: http://abcnews.go.com/Health/GMAHealth/stem-cell-treatment-ease-osteoarthritis-pain-offer-alternative/story?id=13550160 (accessed 4 November 2015).

18. Cyranoski (2010) Available at: http://www.nature.com/news/2010/100817/full/466909a.html; (accessed 4 November 2015).

19. Available at: http://www.regenexx.com/mediaroom/ (accessed 4 November 2015).

20. Available at: http://abcnews.go.com/Health/GMAHealth/stem-cell-treatment-ease-osteoarthritis-pain-offer-alternative/story?id=13550160 (accessed 4 November 2015).

21. Available at: http://www.regenexx.com/regenexx-procedures-family/the-regenexx-procedure-explained/ (accessed 4 November 2015).

22. Available at: http://www.regenexx.com/2009/05/regenexx-in-china/ (accessed 4 November 2015).

23. Available at: http://www.cellmedicinesociety.org/component/content/article/86-news/412-eschenbachwsjresponse (accessed 4 November 2015).

24. Available at: http://www.cellmedicinesociety.org/physicians/international-chapters (accessed 4 November 2015).

25. Available at: http://www.cellmedicinesociety.org/icms-guidelines/basic-tenets (accessed 4 November 2015).

26. Available at: http://www.cellmedicinesociety.org/icms-guidelines/guidelines (accessed 4 November 2015).

27. Available at: http://www.cellmedicinesociety.org/physicians/international-chapters (accessed 4 November 2015).

28. Available at: http://www.cellmedicinesociety.org/home/boards-and-councils/irb (accessed 4 November 2015).

29. Available at: http://www.cellmedicinesociety.org/physicians/irb (accessed 4 November 2015).

30. Continuous review: Available at: http://www.cellmedicinesociety.org/physicians/irb/continuing-review. Reporting of adverse events: Available at: http://www.cellmedicinesociety.org/physicians/irb/sae-reporting (accessed 4 November 2015).

31. Available at: http://www.cellmedicinesociety.org/attachments/184_ICMS%20Open%20Treatment%20Registry%20-%20Overview.pdf (accessed 4 November 2015).

32. Available at: http://www.cellmedicinesociety.org/home/news/press-releases (accessed 4 November 2015).

33. Available at: http://www.cellmedicinesociety.org/home/news/press-releases (accessed 4 November 2015).

34. Available at: http://www.cellmedicinesociety.org/home/news/latest/310-icms-investigates-rnl-bio (accessed 4 November 2015).

35. Available at: http://www.cellmedicinesociety.org/home/news/press-releases/349-world-stemcells-second-participant-in-icms-stemcell-clinic-accreditation-program (accessed 4 November 2015).

36. Available at: http://www.cellmedicinesociety.org/home/news/press-releases/349-world-stemcells-second-participant-in-icms-stemcell-clinic-accreditation-program (accessed 4 November 2015).

37. Available at: http://www.aabb.org/pressroom/pressreleases/pages/pr120530.aspx (accessed 4 November 2015).

38. Available at: http://www.cellmedicinesociety.org/home/news/latest/310-icms-investigates-rnl-bio (accessed 4 November 2015).

39. Available at: http://www.stemcellmx.com/stem-cell-therapy-programs/stem-cell-technology/ (accessed 4 November 2015).

40. Available at: http://www.cellmedicinesociety.org/component/content/article/86-news/407-rmi-accreditation (accessed 4 November 2015).

41. Available at: http://worldstemcells.com/stem-cell-therapy-conditions.html (accessed 4 November 2015).

42. An example of personal testimonies of patients treated for autism can be found at: http://worldstemcells.com/stem-cell-therapy-autism.html (accessed 4 November 2015).

43. Available at: http://www.ipscell.com/2013/11/cell-surgical-network-series-part-3-opinion-piece-on-key-concerns/ (accessed 4 November 2015).

44. Available at: http://www.stemcellsconsulting.com/bios/ (accessed 4 November 2015).
